# Smells like inhibition: The effects of olfactory and visual alcohol cues on inhibitory control

**DOI:** 10.1007/s00213-016-4221-1

**Published:** 2016-03-16

**Authors:** R. L. Monk, J. Sunley, A. W. Qureshi, D. Heim

**Affiliations:** Edge Hill University, St Helens Rd, Ormskirk, L39 4QP UK

**Keywords:** Alcohol, Inhibition, Cues, Olfactory, Context, GNAT

## Abstract

**Rationale:**

How the smell of alcohol impacts alcohol-related thoughts and behaviours is unclear, though it is well-documented that alcohol-related stimuli and environments may trigger these.

**Objectives:**

The current study, therefore, aimed to investigate the priming effects of both visual and olfactory alcohol cues on inhibitory control.

**Method:**

Forty individuals (M age = 23.65, SD = 6.52) completed a go/no-go association task (GNAT) which measured reaction times, response accuracy and false alarm rates whilst being exposed to alcohol-related (or neutral) olfactory and visual cues.

**Results:**

Alcohol-related visual cues elicited lower false alarm rates, slower reaction times and higher accuracy rates relative to neutral pictorial cues. False alarm rates were significantly higher for those exposed to alcohol as opposed to neutral olfactory cues.

**Conclusions:**

By highlighting that exposure to alcohol-related olfactory cues may impede response inhibition, the results indicate that exposure to such stimuli may contribute to the activation of cognitive responses which may drive consumption.

**Electronic supplementary material:**

The online version of this article (doi:10.1007/s00213-016-4221-1) contains supplementary material, which is available to authorized users.

The pairing of the psychological and physiological effects of alcohol consumption with related paraphernalia, people or places can lead to conditioned responses to such stimuli, in the absence of the substance (Rohsenow et al. 1990). The presentation of such stimuli (e.g. the sight of an alcoholic beverage) has been shown to trigger such responses in both clinical and non-clinical populations (e.g. Cooney et al. 1987; Kenny et al. 2006; Nees et al. 2012; Siegel 2001; Traylor et al. 2011; see also Glautier et al. 1992; Kambouropoulos and Staiger 2001; Ramirez et. al. 2014). These include physiological arousal (Kenny 2007; Sinha et al. 2009), such as increased salivation (Rohsenow et al. 1994), electro dermal activity (Garland et al. 2012; Stormark et al. 1993), and heart rate (Ingjaldsson et al. 2003). Exposure to substance-related cues and environments has also been found to be related to changes in alcohol consumption (Monk et al. 2015), as well as related cognitions (Monk and Heim 2013a, b, 2014), relapse (e.g. Carter and Tiffany 1999; Marlatt 1990; Siegel 2005; Zironi et al. 2006) and craving (Conklin and Tiffany 2002; Courtney and Ray 2014; Modell and Mountz 1995).

Such findings are in keeping with the notion that substance-related cues not only involuntarily capture people’s attention but also automatically trigger arousal associations (Field and Cox 2008; Wiers et al. 2002). Accordingly, alcohol-salient environments can be important contextual moderators of attentional biases, as has been demonstrated in both clinical (Field et al. 2014) and non-clinical groups (Albery et al. 2015). For instance, light drinkers are passively exposed to high levels of alcohol-related cues in their everyday lives (e.g. by spending much of their time in bars/pubs), whilst heavy drinkers are actively engaged with the alcohol-related cues in their environment (when drinking). As such, light drinkers display higher levels of attentional bias towards alcohol-related words (passive cues) in comparison to heavy drinkers. Specifically, in contrast to light drinkers, heavy drinkers are actively involved in alcohol consumption, meaning they display high levels of alcohol-related attentional interference, regardless of how much time they spend in alcohol-related contexts. Further attentional interference in response to passive cue exposure is thus not evident (Albery et al. 2015). It is therefore apparent that alcohol-related attentional biases fluctuate and are shaped by exposure to the contextual cues that individuals encounter in everyday life. Context can therefore influence the degree of attentional bias individuals have towards alcohol.

Inhibition controls the strength of alcohol-related attentional biases (Field and Cox 2008) and is one of the processes believed to underlie the aetiology of addictive behaviours (Wiers et al. 2002). Inhibitory control relies on a limited resource (Inzlicht and Berkman 2015; Muraven and Baumeister 2000) which may be overwhelmed in the presence of motivational alcohol cues (although see Monti and Rohsenow 1999 for cue exposure therapy). Indeed, it has been suggested that higher levels of impulsiveness and lower inhibitory control are associated with stronger cue-elicited cravings for alcohol in clinical samples (Papachristou et al. 2013). Changes in inhibitory control responses during exposure to alcohol-related pictorial cues have also been observed. Specifically, in a go/no-go task, participants appear to make more commission errors (false alarm rate; FAR) when no-go stimuli are super-imposed on alcohol-related images (Petit et al. 2012). Further, participants seem to respond faster when alcohol stimuli are ‘go’ stimuli (Kreusch et al. 2013).

However, whilst research has focused on the way in which alcohol-related visual, auditory and tactile cues shape alcohol-related thoughts and behaviours (Stein et al. 2000), there has been a distinct lack of exploration into possible effects of olfactory stimuli. Early research indicates that the smell of alcohol leads to an increase in self-reported desire to drink (Laberg 1990), whilst smelling an alcoholic drink appears to increase reported craving (Litt and Cooney 1999). Whilst a review by Schacht and colleagues (2013) notes the use of olfactory cues in studies of alcohol-related cue reactivity, the impact of olfaction on inhibitory processes remains unexplored. The current research therefore aims to examine this suggestion by introducing olfactory cues during the administration of the go/no-go task (GNG; Nosek and Banaji 2001).

It was predicted that alcohol-related (as opposed to neutral) olfactory cues would make alcohol-related stimuli more salient, leading to difficulties in inhibiting responses and resulting in higher FARs for these no-go alcohol-related stimuli.

## Method

### Participants

Forty participants (21 females, aged 19–48 years, M = 22.76, SD = 5.97, 19 males, aged 19–52 years, M = 24.63, SD = 7.11*)* were recruited via opportunity sampling and were randomly allocated to either alcohol (*n* = 20) or neutral olfactory cue conditions. Preliminary analyses suggested that there were no significant differences in the age (*t* (38) = .49, *p* = 0.63), gender (*Χ*^2^ (1, *N* = 40) = 0.09, *p* = 0.77) or AUDIT scores (*t* (38) = −0.33, *p* = 0.75) of those randomly allocated to the alcohol or control olfactory conditions. Demographics for the groups are shown in Table 1, as well as mean AUDIT scores. The latter are slightly above the cut-off for clinical assessment (scores of 8 or above being deemed to indicate hazardous or harmful alcohol use; Babor et al. 2001; Saunders et al. 1993). Participants are hence comparable with recent research using UK student samples (Clarke et al. 2015; Moss et al. 2015).

**Table 1:** Mean and standard deviations for age and AUDIT values, *N* for gender, for olfactory cue conditions

	Alcohol smell	Neutral smell
Age	24.56 (7.88)	23.21 (6.99)
AUDIT*	9.75 (7.40)	10.50 (7.05)
Gender (*N*)	9 M, 11 F	10 M, 10 F

*Alcohol cue group range 0–28, and neutral cue range 1–29. *Boxplots* reveal no outliers

### Design

A 2 (visual stimuli: alcohol and neutral) × 2 (olfactory cues: alcohol or neutral) mixed-groups design was used to examine the effect of olfactory and visual cues on FARs, reaction time on go trials (ms), and accuracy on the GNAT. All participants were exposed to both alcohol and neutral visual stimuli and random allocation was used to allocate participants to the olfactory cueing or control conditions.

### Stimuli and materials

The Alcohol Use Disorder Identification Test (AUDIT—Saunders et al. 1993) is a 10-item questionnaire which explores the domains of alcohol consumption, drinking behaviour and alcohol-related problems. Responses to each question are scored from 0 to 4, with a maximum possible score of 40. AUDIT provides a simple method of early detection of hazardous and harmful alcohol use in primary health care settings and is derived from a cross-national study. Good internal consistency on this measure was demonstrated in the current sample (Cronbach’s *α* = 0.72).

The go/no-go association task (GNAT) used in this research utilised two picture sets for the visual cues: one set contained neutral pictures (the letter K vs. the other 25 letters) and the other set with bar-related pictures (a beer bottle vs. 25 water bottle pictures). Pictures of the letter K and beer were the target stimuli (14 % were no-go—36 no-go, 224 go stimuli used). All pictures were graphically equivalent in terms of colours, contrasts, objects shown and viewing angles. Branding was removed as appropriate and all pictures were presented on a white background using E-Prime 2.0 software.

A pre-treated olfactory mask was worn by all participants. This design was preferred to more traditional olfactory research designs in which participants inhale from a beaker, as it has been suggested that this earlier design may produce demand characteristics in responses (Litt and Cooney 1999). Participants in the current study were informed that external light, sound and smell have been previously demonstrated to adversely impact performance in the GNAT. The task instructions then went on to explain that it was for this reason that the current study had taken steps to control these factors, by using a mask, controlled lighting and sound-cancelling headphones which respondents were also required to wear. This was done with the intention of making the olfactory cues less overt so as to prevent demand characteristics. Post-test assessments suggested that although participants had noticed a slight smell from the mask, they had not inferred the true aim of the research nor interpreted smell as a variable of interest in the research. During debriefing, participants were also asked not to share the aims of the research with other potential study candidates.

Research indicates that an odour that is not from the same semantic category as alcohol, but is equally liked or disliked, will ensure a priming effect (Smeets and Dijksterhuis 2014). Furthermore, olfactory intensity has been shown to impact odour perception, specifically at higher concentrations (Smeets and Dijksterhuis 2014). It was therefore necessary to ensure that the filters inside the masks worn by participants contained subtle yet comparable scents. Pilot testing was, therefore, carried out using a number of varying alcohol-related and neutral smells (at varying doses) in order to uncover two scents (one from each category) which were equally liked and rated as having an equivalent intensity. A pipette was utilised in order pre-treat the masks with small amounts of vodka (5 ml of diluted Glenn’s vodka, 1:5 dilution, administered as the alcohol-related olfactory cue) or citrus oil (5 ml of diluted oil, 1:10 dilution, as the control condition).

### Procedure

This research was approved by the appropriate ethics committee and the research was therefore been performed in accordance with the ethical standards laid down in the 1964 Declaration of Helsinki. All persons gave their informed consent prior to their inclusion in the study. This research introduced olfactory cues into the paradigm of Kreusch et al. (2013), who inserted alcohol-related visual into the GNAT. Following ethical approval, participants were recruited, briefed and seated in front of a computer. They were then asked to fit the inhalation mask.

**Fig. 1:**
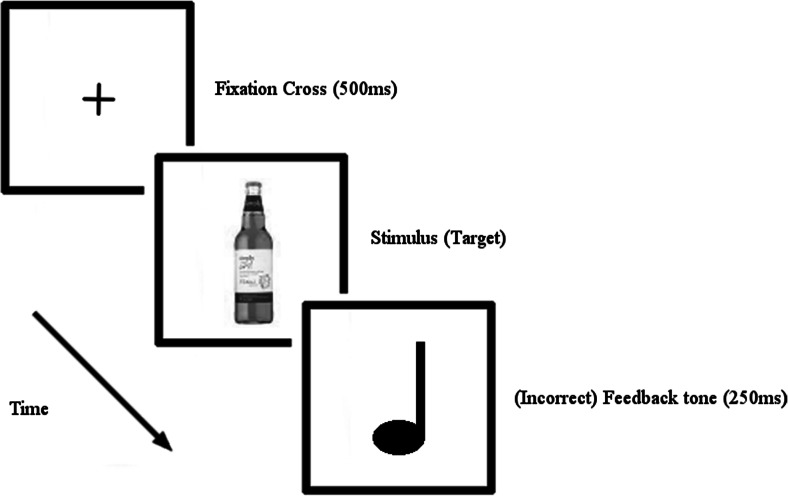
Go/no-go trial procedure

Response inhibition towards alcohol cues was assessed by a GNAT programmed with E-Prime 2.0. In the GNAT, each trial began with a white fixation cross on a black background for 500 ms. Immediately, after the onset of the fixation cross, a stimulus was presented in the centre against the black screen for 500 ms Fig. 1.

Participants were required to inhibit their response to target stimuli (see materials; alcohol condition = bottle of beer; neutral condition = letter K), but to respond to all other stimuli. If participants responded incorrectly (i.e. they pressed the space bar when a target stimuli was presented in no-go trials), a feedback tone (250 ms) was presented. The experiment was organised into 16 blocks, eight with alcohol visual stimuli and eight with neutral (letter) stimuli. The order of blocks was randomised and trial order was pseudo-randomised (with no more than three of any given trial being permitted in a row and no blocks starting with a no-go trial). The distribution of the blocks and trials was also checked post hoc in order to ensure distribution equivalency between olfactory and neutral cue conditions. The study lasted approximately 30 min and included breaks between blocks to mitigate fatigue. The mask was removed at the end of testing. Following the GNAT task, participants were asked to provide basic demographic information and to complete the AUDIT, prior to receiving a full debrief. This was the final component of testing in order to limit the signal strength of the study (c.f. Davies and Best 1996).

## Results

Missing trial data accounted for only 4 % (1371/31201). A low pass filter (200 ms) was used, as stimuli were presented for 500 ms, with the experiment moving onto the next trial at that point. Following screening for normality, a 2 (visual stimuli: alcohol and neutral) × 2 (olfactory cues: alcohol or non-alcohol) mixed Factorial ANOVA was used to examine the effect of olfactory and visual stimuli on a go/no-go task. Three analyses were conducted: one with FAR as the dependent variable, one with reaction times on go trials (ms), and one with response accuracy on Go trials.^1^ Means and standard deviations of participants’ FAR, accuracy and reaction times across olfactory and visual cueing conditions are reported in Table 2. A main effect of olfactory cue was found (*F*(1, 38) = 5.42, *p* = 0.03, *η*_*p*_^2^ = 0.13) such that the FAR was higher for those receiving the alcohol olfactory cue (M = 0.56, SD = 0.04) than for the control olfactory cue (M = 0.42, SD = 0.04). Furthermore, there was a main effect of pictorial target (*F*(1, 38) = 15.65, *p* ≤ 0.01, *η*_*p*_^2^ = 0.29), such that the FAR was higher for the neutral (letters) pictorial targets in comparison with the alcohol pictorial targets. There was no significant interaction between olfactory cue and pictorial target (*F*(1, 38) = 0.29, *p* = 0.60, *η*_*p*_^2^ = 0.01).

**Table 2:** Mean and standard deviations for false alarm rates, reaction time and accuracy for olfactory and pictorial cue conditions

	Alcohol picture			Neutral picture		
	FAR	RT	ACC	FAR	RT	ACC
Alcohol smell	0.51 (0.24)	272.62 (43.16)	0.94 (0.09)	0.60 (0.18)	287.71 (41.21)	0.92(0.10)
Neutral smell	0.36 (0.18)	254.13 (57.63)	0.96 (0.04)	0.48 (0.19)	293.03 (99.50)	0.95 (0.07)

*FAR* false alarm rate, *RT* reaction time *ACC* accuracy to go trials

### Reaction time on go trials

Whilst there was no main effect of olfactory cue on reaction time (*F*(1, 38) = 0.16, *p* > 0.69, *η*_*p*_^2^ = 0.00), a significant main effect of pictorial target on reaction time was found (*F*(1, 38) = 4.77, *p* = 0.04, *η*_*p*_^2^ = 0.11) such that reaction time was slower for the neutral pictorial target than for the alcohol pictorial target. There was no significant interaction between olfactory cue and the pictorial target (*F*(1, 38) = 0.93, *p* = 0.34, *η*_*p*_^2^ = 0.02).

### Accuracy on go trials

There was no main effect of olfactory cue on go accuracy rates (*F*(1, 38) = 0.76, *p* = 0.39, *η*_*p*_^2^ = 0.02) with the accuracy slightly higher for the control olfactory cue than the alcohol olfactory cue. Yet there was a main effect of pictorial target on accuracy (*F*(1, 38) = 4.26, *p* = 0.05, *η*_*p*_^2^ = 0.01), such that accuracy was significantly higher for the alcohol pictorial target than the neutral pictorial target. There was no significant interaction between olfactory cue and pictorial target (*F*(1, 38) = 0.71, *p* = 0.41, *η*_*p*_^2^ = 0.02).

Separate analyses for alcohol and neutral visual stimuli were also run (Appendix 1). The pattern shown was the same as for combined analyses. D′ scores were also calculated^2^ (as per Macmillan and Creelman 1991). Combined analyses showed the same pattern of results as for FAR, as did separate analyses (Appendix 1). The separate analyses were conducted due to the different processes involved in letter recognition and selection of pictures (Carr et al. 1982).

## Discussion

The aim of the present research was to examine the extent to which olfactory cues impact response inhibition in a population of social-drinkers using GNAT. As hypothesised, FARs were significantly higher among respondents receiving the alcohol olfactory cue than those in the control condition. Nonetheless, that this effect was generalised across both alcohol and non-alcohol-related visual cues was not predicted. Early research indicates that the smell of alcohol leads to increases in self-reported desire to drink (Laberg 1990) and craving (Litt and Cooney 1999). The current research contributes to this body of knowledge by suggesting that inhibitory control may also be affected by the smell of alcohol in a similar fashion. It may be hypothesised that this operates via the triggering of associated physiological and cognitive processes, in-line with theories of cue reactivity. These findings are also apparently in-line with the assertion that (non-olfactory) cues elicit a psychomotor-activating response (c.f. Wiers et al. 2002). This may lead to difficulties in inhibiting a dominant response (also see Roberts et al. 2014), thus leading to more errors than in those not exposed to such cues. In this way, the current findings support the findings of previous research that exposure to alcohol-related stimuli results in reduced accuracy in tasks requiring inhibitory control (Petit et al. 2012).

However, it was not expected that alcohol-related olfactory cues would affect inhibition (manifest in increased FARs), irrespective of the type of visual cue. These findings may therefore demonstrate a pattern of generalised response impairment (i.e. inhibition impairment regardless of visual stimuli). This effect is in keeping with the wider literature that suggests that responses to unique stimuli (e.g. Baldi et al. 2004; Mühlberger et al. 2014) including olfactory cues (e.g. Daly et al. 2001), can become generalised to wider contexts and stimuli. In other words, there may be a carryover effect from responding to specific cues, meaning that responses translate to wider stimuli. Whilst not hitherto examined in this field, such findings may therefore suggest that alcohol-related olfactory cues may reduce inhibition to both alcohol-related and non-alcohol-related cues. The real-world drivers of processes such as attentional bias and inhibitory control require further examination. Even so, the current research contributes to the growing body of research that proffers the role of a myriad of complex contextual cues.

Nonetheless, it should be noted that there were no observed effects of olfactory cue on response time or accuracy on go trials. In the GNAT, response selection occurs in conjunction with response inhibition, as respondents must select whether to execute an appropriate response or to inhibit an inappropriate response (Suskauer et al. 2008). If one considers that response selection is needed to process which responses to make, then response inhibition is needed to withhold the response for ‘no-go’ target (see below for further discussion). If the olfactory cue impacted selection (rather than or more than response inhibition) then it would be expected that performance on the task using letters would be impaired over and above performance on the alcohol-related pictorial stimuli, as the former arguably places a greater demand on response selection. The current findings may therefore indicate that olfactory cues may have very specific effects on response inhibition (rather than selection). Such assertions are, nonetheless, speculative at this stage and further research is recommended to test this hypothesis.

Hypotheses regarding the effect of visual alcohol-related cues were not supported. Here, FAR and accuracy was higher, and reaction times slower, for the neutral stimuli in comparison with responses to alcohol-related pictures. Such findings do not align with previous indications of heightened response times and cue reactivity towards alcohol stimuli (Kreusch et al. 2013; Petit et al. 2012). Petit et al. (2012) also showed that participants found it harder to inhibit responses to alcohol-related stimuli, leading to reduced accuracy. These divergent results may be explained by a number of differences between the stimuli used in the current study compared to others. First, Kreusch et al. (2013) used pictures of neutral objects (e.g. a stapler) as the non-target stimuli, whereas the current study used letters, which are of a different semantic category and thus may evoke different responses and or processes in responding. Second, there were more non-target stimuli in the current study when compared to previous research. This may require a greater degree of response selection from participants, in addition to the response inhibition necessitated by this task. As response selection involves the selection of either the appropriate response or the choice to inhibit an inappropriate response (Simmonds et al. 2008), tasks requiring response selection as well as response inhibition may result in slower responses and greater chances of errors due to additional processing. Furthermore, as noted by Kreusch and colleagues (2013), the provision of alcohol-related questions prior to testing may have primed participants, whilst in this study this was avoided. Further research is required to test such assertions.

It should be noted that, in the current research, there was no inclusion of a non- odour condition. That the citrus scent could have had an independent effect over the alcohol olfactory cue can thus not be excluded (Smeets and Dijksterhuis 2014). Further research in this regard may therefore be recommended. It is also suggested that other scents should be tested in the future, in order to assess which types of alcohol elicit the greatest response (c.f. Schneider et al. 2001 on beer). The expansion of this research beyond a purely student-based sample is also recommended, given that University students are immersed in a social, pub-based drinking culture (Borsari and Carey 2001; Karam et al. 2007; Straus and Bacon 1953). Context-related cueing may therefore be particularly likely (c.f. Rumelhart and Todd 1993). It is also advisable that future research test trait levels of impulsivity during testing, as baseline variability in impulsiveness between the alcohol and neutral olfactory conditions cannot presently be ruled out. Whilst the random allocation of participants to olfactory condition reduces this possibility, such potential does require acknowledgment.

Finally, it is recommended that further research is required before firmer claims can be made as the effect of alcohol-related visual cues on inhibition. This is recommended in light of two potential limitations of the current research. First, reverse instruction blocks were not inserted into the current GNAT paradigm (i.e. where identical stimuli are assigned as both go targets or no-go distracters in randomly administered trials). Whilst recent research has shown little variation in results when reversed conditions are included within an alcohol-related GNAT (c.f. Pennington et al. in press), the exploration of reversed conditions response patterns would add further weight to the current findings. Specifically, a recent review has suggested that the valence of stimuli can affect the selection of appropriate or inappropriate actions, with possible implications for impulsivity and addiction (Guitart-Masip et al. 2014). Including a reversed condition, where responses are only made to target stimuli (alcohol-related or letter K) may therefore have allowed for deductions regarding whether the effects were primarily due to generalised response invigoration or inhibitory deficits. Second, there were inherent variations in task difficulty within the current tasks: In the first task, the participants must select an alcohol no-go target among non-alcohol visual distracters. In the second task, they must select a K-letter no-go target among other letter visual distracters (a potentially more difficult task). This means that further research is necessary in order to disentangle the effects of task difficult and stimuli type on response times, accuracy and reaction time. Nevertheless, the main finding, that olfactory cues affected such measures, irrespective of visual cues, is a novel and important research finding worthy of further consideration.

Overall, this research offers an original insight into the importance of acknowledging olfactory alcohol cues in developing a comprehensive understanding of alcohol-related behaviour. Context-related reductions in inhibitory control may lead to increases in consumption, or to relapse in abstinence users.

## Electronic supplementary material

Below is the link to the electronic supplementary material.

ESM 1 (DOCX 13 kb)

## Footnotes

1 FAR to no-go stimuli are commission errors to K or alcohol images—when participants are not meant to respond but do. The ‘hit rate’ to Go trials is equivalent to omission errors (proportion of stimuli responded to correctly)

2 **Combined analyses**: There was a main effect of olfactory cue (*F*(1, 38) = 4.66, *p* = 0.04, *η*_*p*_^2^ = 0.11), insomuch that D’ for those receiving the alcohol cue was lower (M = 1.66, SD = 0.61) than those receiving the neutral cue (M = 2.00, SD = 0.62), indicating performance was significantly affected by the alcohol olfactory cue. There was also a main effect of pictorial target (*F*(1, 38) = 30.76, *p* ≤ 0.01, *η*_*p*_^2^ = 0.45), with D′ for alcohol stimuli higher (M = 2.06, SD = 0.67) than for neutral stimuli (M = 1.60, SD = 0.51), indicating performance on the neutral stimuli was significantly worse than for alcohol stimuli. There was no interaction between olfactory cue and pictorial target (*F*(1, 38) = 0.17, *p* = 0.68, *η*_*p*_^2^ = 0.00). **Separate analyses:** For the alcohol stimuli, there was a marginal main effect of olfactory cue (*F*(1, 38) = 3.48, *p* = .07, *η*_*p*_^2^ = 0.08), insomuch that D’ for those receiving the alcohol cue was lower (M = 1.87, SD = 0.66) than those receiving the neutral cue (M = 2.25, SD = 0.63). For the neutral stimuli, there was also a marginal main of effect of olfactory cue (*F*(1, 38) = 4.10, *p* = 0.05, *η*_*p*_^2^ = 0.10), with D’ for those receiving the alcohol cue lower (M = 1.44, SD = 0.48) than those receiving the neutral cue (M = 1.75, SD = 0.50). Overall, this suggests that performance for both types of stimuli was affected by the alcohol olfactory cue.

## References

[CR1] Albery IP, Sharma D, Noyce S, Frings D, Moss AC (2015). Testing a frequency of exposure hypothesis in attentional bias for alcohol-related stimuli amongst social drinkers. Addict Behav Rep.

[CR2] Babor, T. F., Higgins-Biddle J. C., Saunders, J. B., Monteiro, M. G. (2001). The alcohol use disorders identification test, guidelines for use in primary care, Second Edition, Department of Mental Health and Substance Dependence, World Health Organization.

[CR3] Baldi E, Lorenzini CA, Bucherelli C (2004). Footshock intensity and generalization in contextual and auditory-cued fear conditioning in the rat. Neurobiol Learn Mem.

[CR4] Borsari B, Carey KB (2001). Peer influences of college drinking: a review of the research. J Subst Abus.

[CR5] Carr TH, McCauley C, Sperber RD, Parmelee CM (1982). Words, pictures, and priming: on semantic activation, conscious identification, and the automaticity of information processing. J Exp Psychol Hum Percept Perform.

[CR6] Carter BL, Tiffany ST (1999). Meta-analysis of cue-reactivity in addiction research. Addiction.

[CR7] Clarke NC, Field M, Rose AK (2015). Evaluation of a brief personalised intervention for alcohol consumption in college students. PLoS One.

[CR8] Conklin CA, Tiffany ST (2002). Applying extinction research and theory to cue exposure addiction treatments. Addiction.

[CR9] Cooney NL, Gillespie RA, Baker LH, Kaplan RF (1987). Cognitive changes after alcohol cue exposure. J Consult Clin Psychol.

[CR10] Courtney KE, Ray LA (2014). Subjective responses to alcohol in the lab predict neural responses to alcohol cues. J Stud Alcohol Drugs.

[CR11] Daly KC, Chandra S, Durtschi ML, Smith BH (2001). The generalization of an olfactory-based conditioned response reveals unique but overlapping odour representations in the moth Manduca sexta. J Exp Biol.

[CR12] Davies JB, Best DW (1996). Demand characteristics and research into drug use. Psychol Health.

[CR13] Field M, Cox WM (2008). Attentional bias in addictive behaviors: a review of its development, causes, and consequences. Drug Alcohol Depend.

[CR14] Field M, Marhe R, Franken IH (2014). The clinical relevance of attentional bias in substance use disorders. CNS Spectr.

[CR15] Garland EL, Franken IH, Sheetz JJ, Howard MO (2012). Alcohol attentional bias is associated with autonomic indices of stress-primed alcohol cue-reactivity in alcohol-dependent patients. Exp Clin Psychopharmacol.

[CR16] Glautier S, Drummond DC, Remington B (1992). Different drink cues elicit different physiological responses in non-dependent drinkers. Psychopharmacology.

[CR17] Guitart-Masip M, Duzel E, Dolan R, Dayan P (2014). Action versus valence in decision making. Trends Cogn Sci.

[CR18] Ingjaldsson JT, Thayer JF, Laberg JC (2003). Craving for alcohol and pre-attentive processing of alcohol stimuli. Int J Psychophysiol.

[CR19] Inzlicht M, Berkman E (2015). Six questions for the resource model of self-control (and some answers). Soc Personal Psychol Compass.

[CR20] Karam E, Kypri K, Salamoun M (2007). Alcohol use among college students: an international perspective. Curr Opin Psychiatry.

[CR21] Kambouropoulos N, Staiger PK (2001). The influence of sensitivity to reward on reactivity to alcohol-related cues. Addiction.

[CR22] Kenny PJ (2007). Brain reward systems and compulsive drug use. Trends Pharmacol Sci.

[CR23] Kenny KJ, Chen SA, Kitamura O, Markou A, Koob GF (2006). Withdrawal drives heroin consumption and decreases reward sensitivity. J Neurosci.

[CR24] Kreusch F, Vilenne A, Quertemont E (2013). Response inhibition toward alcohol-related cues using an alcohol go/no-go task in problem and non-problem drinkers. Addict Behav.

[CR25] Laberg JC (1990). What is presented, and what prevented, in cue exposure and response prevention with alcohol dependent subjects?. Addict Behav.

[CR26] Litt MD, Cooney NL (1999). Inducing craving for alcohol in the laboratory. Alcohol Res Health.

[CR27] Macmillan NA, Creelman CD (1991). Detection theory: a user’s guide.

[CR28] Marlatt GA (1990). Cue exposure and relapse prevention in the treatment of addictive behaviours. Addict Behav.

[CR29] Modell JG, Mountz JM (1995). Focal cerebral blood flow change during craving for alcohol measured by SPECT. J Neuropsychiatry Clin Neurosci.

[CR30] Monk RL, Heim D (2013). A critical systematic review of alcohol-related outcome expectancies. Subst Use Misuse.

[CR31] Monk RL, Heim D (2013). Panoramic projection: affording a wider view on contextual influences on alcohol-related cognitions. Exp Clin Psychopharmacol.

[CR32] Monk RL, Heim D (2013). Environmental context effects on alcohol-related outcome expectancies, efficacy and norms: a field study. Psychol Addict Behav.

[CR33] Monk RL, Heim D (2014). A real-time examination of context effects on alcohol cognitions. Alcohol Clin Exp Res.

[CR34] Monk, R.L., Heim, D., Qureshi, A., Price, A. (2015). “I have no clue what I drunk last night” using smartphone technology to compare in-vivo and retrospective self-reports of alcohol consumption. PLoS ONE 10(5):e0126209 10.1371/journal.pone.0126209 PMC4437777 25992573

[CR35] Monti PM, Rohsenow DJ (1999). Coping-skills training and cue-exposure therapy in the treatment of alcoholism. Alcohol Res Health.

[CR36] Moss AC, Spada MM, Harkin J, Albery IP, Rycroft N, Nikčević AV (2015). ‘Neknomination’: predictors in a sample of UK university students. Addict Behav Rep.

[CR37] Mühlberger A, Andreatta M, Ewald H, Glotzbach-Schoon E, Tröger C, Baumann C, Pauli P (2014). The BDNF Val66Met polymorphism modulates the generalization of cued fear responses to a novel context. Neuropsychopharmacology.

[CR38] Muraven M, Baumeister RF (2000). Self-regulation and depletion of limited resources: does self-control resemble a muscle?. Psychol Bull.

[CR39] Nees F, Diener C, Smolka MN, Flor H (2012). The role of context in the processing of alcohol-relevant cues. Addict Biol.

[CR40] Nosek BA, Banaji MR (2001). The go/no-go association task. Soc Cogn.

[CR41] Papachristou H, Nederkoorn C, Havermans R, Bogngers P, Beunen S, Jansen A (2013). Higher levels of trait impulsiveness and a less effective response inhibition are linked to more intense cue-elicited craving for alcohol in alcohol-dependent patients. Psychopharmacology (Berl).

[CR42] Pennington, C. R., Qureshi, A., Monk, R. L., Heim, D. (in press). The effects of stereotype threat and contextual cues on alcohol users’ inhibitory control. Addictive Behaviors 10.1016/j.addbeh.2015.11.014 26657819

[CR43] Petit G, Kornreich C, Noël X, Verbanck P, Campanella S (2012). Alcohol-related context modulates performance of social drinkers in a visual Go/No-Go task: a preliminary assessment of event-related potentials. PLoS One.

[CR44] Ramirez JJ, Monti PM, Colwill RM (2014). Alcohol-cue exposure effects on craving and attentional bias in underage college-student drinkers. Psychol Addict Behav.

[CR45] Roberts W, Miller MA, Weafer J, Fillmore MT (2014). Heavy drinking and the role of inhibitory control of attention. Exp Clin Psychopharmacol.

[CR46] Rohsenow DJ, Niaura RS, Childress AR, Abrams DB (1990). Cue reactivity in addictive behaviours: theoretical and treatment implications. Int J Addict.

[CR47] Rohsenow DJ, Monti PM, Rubonis AV, Sirota AD, Niaura RS, Colby SM, Abrams DB (1994). Cue reactivity as a predictor of drinking among male alcoholics. J Consult Clin Psychol.

[CR48] Rumelhart DE, Todd PM, Meyer DE, Kornblum S (1993). Learning and connectionist representations. Attention and performance XIV: synergies in experimental psychology, artificial intelligence, and cognitive neuroscience.

[CR49] Saunders JB, Aasland OG, Babor TF, De la Fuente JR, Grant M (1993). Development of the alcohol use disorders identification test (AUDIT). WHO collaborative project on early detection of persons with harmful alcohol consumption-II. Addiction.

[CR50] Schacht JP, Anton RF, Myrick H (2013). Functional neuroimaging studies of alcohol cue reactivity: a quantitative meta-analysis and systematic review. Addict Biol.

[CR51] Schneider F, Habel U, Wagner M, Franke P, Salloum JB, Shah NJ, Zilles K (2001). Subcortical correlates of craving in recently abstinent alcoholic patients. Am J Psychiatry.

[CR52] Siegel S (2001). Pavlovian conditioning and drug overdose: when tolerance fails. Addict Res Theory.

[CR53] Siegel S (2005). Drug tolerance, drug addiction, and drug anticipation. Curr Dir Psychol Sci.

[CR54] Simmonds DJ, Pekar JJ, Mostofsky SH (2008). Meta-analysis of Go/No-go tasks demonstrating that fMRI activation associated with response inhibition is task-dependent. Neuropsychologia.

[CR55] Sinha R, Fox HC, Hong KA, Bergquist K, Bhagwagar Z, Siedlarz KM (2009). Enhanced negative emotion and alcohol craving, and altered physiological responses following stress and cue exposure in alcohol dependent individuals. Neuropsychopharmacology.

[CR56] Smeets MAM, Dijksterhuis GB (2014). Smelly primes—when olfactory primes do or do not work. Front Psychol.

[CR57] Stein KD, Goldman MS, Del Boca FK (2000). The influence of alcohol expectancy priming and mood manipulation on subsequent alcohol consumption. J Abnorm Psychol.

[CR58] Stormark KM, Laberg JC, Bjerland T, Hugdahl K (1993). Habituation of electrodermal reactivity to visual alcohol stimuli in alcoholics. Addict Behav.

[CR59] Straus R, Bacon S (1953). Drinking in college.

[CR60] Suskauer SJ, Simmonds DJ, Fotedar S, Blankner JG, Pekar JJ, Denckla MB, Mostofsky SH (2008). Functional magnetic resonance imaging for abnormalities in response selection in attention deficit hyperactivity disorder: differences in activation associated with response inhibition but not habitual motor response. J Cogn Neurosci.

[CR61] Traylor AC, Parrish DE, Copp HL, Bordnick PS (2011). Using virtual reality to investigate complex and contextual cue reactivity in nicotine dependent problem drinkers. Addict Behav.

[CR62] Wiers RW, van Woerden N, Smulders FT, de Jong PJ (2002). Implicit and explicit alcohol-related cognitions in heavy and light drinkers. J Abnorm Psychol.

[CR63] Zironi I, Burattini C, Aicardi G, Janak PH (2006). Context is a trigger for relapse to alcohol. Behav Brain Res.

