# Supplementary material for: Smells like inhibition: The effects of olfactory and visual alcohol cues on inhibitory control
Source: Psychopharmacology (Berl). 2016 Mar 16;233:1331–7. doi: 10.1007/s00213-016-4221-1 (PMC4819591; doi:10.1007/s00213-016-4221-1)
Supplement: Supplementary file 1 — (DOCX 13 kb) [file 213_2016_4221_MOESM1_ESM.docx]

Separate analyses of alcohol and neutral stimuli

False Alarm Rate (FAR)

Alcohol stimuli

There was a main effect of olfactory cue (*F*(1, 38) = 4.87, *p* =.03,
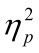
= .11), insomuch that the FAR for those receiving the alcohol cue was higher (M= .51, SD= .24) than those receiving the neutral cue (M= .36, SD= .18).

Neutral stimuli

There was a main effect of olfactory cue (*F*(1, 38) = 4.30, *p* =.05,
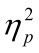
= .10), insomuch that the FAR for those receiving the alcohol cue was higher (M= .60, SD= .18) than those receiving the neutral cue (M= .47, SD= .19).

Go RT

Alcohol stimuli

There was no main effect of olfactory cue (*F*(1, 38) = 1.32, *p* =.26,
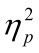
= .03), insomuch that the FAR for those receiving the alcohol cue was similar (M= 272.62, SD= 43.16) to those receiving the neutral cue (M= 254.13, SD= 57.63).

Neutral stimuli

There was no main effect of olfactory cue (*F*(1, 38) = 0.05, *p* =.83,
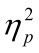
= .00), insomuch that the FAR for those receiving the alcohol cue was similar (M= 287.71, SD= 41.21) to those receiving the neutral cue (M= 293.03, SD= 99.50).

Go Accuracy

Alcohol stimuli

There was no main effect of olfactory cue (*F*(1, 38) = 0.37, *p* =.55,
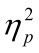
= .01), insomuch that the FAR for those receiving the alcohol cue was similar (M= .94, SD= 0.09) to those receiving the neutral cue (M= .96, SD= .04).

Neutral stimuli

There was no main effect of olfactory cue (*F*(1, 38) = 1.09, *p* =.30,
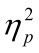
= .03), insomuch that the FAR for those receiving the alcohol cue was similar (M= .92, SD= .10) to those receiving the neutral cue (M= .95, SD= 0.03).
